# A Review of Online Dyslexia Learning Modules

**DOI:** 10.3389/feduc.2020.00118

**Published:** 2020-07-14

**Authors:** Alida Anderson, Gabrielle L. Sarlo, Hannah Pearlstein, Lauren M. McGrath

**Affiliations:** 1 School of Education, Special Education: Learning Disablities Program, American University, Washington, DC, United States; 2 Department of Psychology, American University, Washington, DC, United States; 3 University of Michigan, Ann Arbor, MI, United States; 4 Department of Psychology, University of Denver, Denver, CO, United States

**Keywords:** dyslexia, neuromyths, learning modules, teacher education, neuroscience and education, public education and awareness

## Abstract

This paper presents a comprehensive review of publicly available online dyslexia learning modules with a particular focus on the extent to which modules address the prevalent myth that dyslexia is caused by “backwards reading.” The authors conducted a systematic internet search to identify publicly available online dyslexia learning modules and coded the content across education, neurocognition, and policy disciplinary domains. We identified 18 topics across a small number (*N* = 14) of publicly available modules that focused on dyslexia, with only two modules directly addressing this dyslexia myth. While both identified this myth as false, neither provided information about the neurocognitive underpinnings of dyslexia to explain why this myth is false. This review will be useful for guiding further development of online dyslexia learning modules which are urgently needed due to persisting misinformation about this disorder. The coded content reviews of each module will also be beneficial for directing attention to existing resources for professional development on dyslexia.

## INTRODUCTION

Dyslexia is one of the most prevalent disorders of childhood and the most prevalent form of specific learning disability (Cortiella and Horowitz, 2014). Nearly five decades of research has led to a consensus definition of dyslexia, which has been adopted by the National Institutes of Child Health and Development (NICHD) and the International Dyslexia Association (IDA) (Lyon et al., 2003). The consensus definition describes primary difficulties in accurate and/or fluent word recognition and highlights underlying phonological difficulties that lead to word recognition difficulties for many children (Lyon et al., 2003). Dyslexia is a neurobiological disorder with both genetic and environmental influences (Pennington and Olson, 2005). Brain imaging data have revealed structural and functional differences in the brains of individuals with dyslexia (Norton et al., 2015). Impairments in cognitive-linguistic abilities, such as phonological awareness and rapid automatized naming (RAN) are commonly observed in dyslexia (Wolf and Bowers, 1999; Pennington and Lefly, 2001; Vellutino et al., 2004).

Research clearly has shown that the strongest cognitive-linguistic predictor of dyslexia is phonological awareness (Vellutino et al., 2004). Phonological awareness refers to the ability to identify and manipulate the sound structure of language, including the smallest units, phonemes. Phonological awareness is a critical early skill for learning letter-sound correspondences and facilitates early literacy abilities. Research has shown that decoding skills in individuals with dyslexia can be improved through phonological interventions (Torgesen et al., 2001; Shaywitz et al., 2004; Torgesen, 2005) and that intervention response is associated with structural and functional brain alterations in children (Simos et al., 2007; Spironelli et al., 2010; Krafnick et al., 2011) and adults (Eden et al., 2004) with dyslexia.

Remarkably, despite this scientific consensus about the definition and neurobiological and cognitive-linguistic underpinnings of dyslexia, a prevalent myth that dyslexia is caused by seeing letters or words backwards or “backwards reading” persists. We will refer to this myth as the “dyslexia myth” because it is the most prevalent misunderstanding about dyslexia. We acknowledge that there are other widespread misunderstandings about dyslexia that also require attention (e.g., White et al., 2020). The goal of this review is to identify and characterize the content of existing online dyslexia learning modules and to assess the extent to which these existing resources dispel the prevalent myth about backwards reading in dyslexia.

### Dyslexia Myth of “Backwards Reading”

How did this visual myth about letter and word reversals arise and why does it persist? Historically, dyslexia was described first as “word blindness” in individuals who had otherwise typical intelligence but who could not learn to read (Morgan, 1896; Orton, 1925). Early 20th century theories of dyslexia focused on visual-perceptual, visual-memory, and visual-motor problems, forming the basis for the belief that the defining symptom of dyslexia was reading and writing letters and words backwards (see Lilienfeld et al., 2010 for a discussion). The problem is that these theories were rejected over time as the science of dyslexia developed. In fact, a large body of evidence dating back to the 1970s has discredited such basic “visual” theories of dyslexia (e.g., Liberman et al., 1971; Fowler et al., 1977; Fischer et al., 1978; Vellutino, 1979; Ziegler et al., 2010; Treiman et al., 2014).

The dyslexia myth of “backwards reading” persists as a remnant of the visually based theories about reading (see Lilienfeld et al., 2010 for a discussion). This myth has been resistant to change likely due to the complexity of research on dyslexia coupled with a lack of venues for disseminating increasingly complicated representations of science to the public. There is also the problem that some children with dyslexia do make letter reversals in their writing, which serves to reinforce the notion of “backwards reading.” However, letter reversals are developmentally common during early literacy acquisition in typically developing children as well (Vellutino, 1979) and such reversals early in literacy acquisition (i.e., kindergarten) are unrelated to later reading abilities (i.e., grades 2,3) (Treiman et al., 2014). Long-standing lines of research have shown that children with dyslexia do not show visual-spatial weaknesses with non-linguistic stimuli, such as geometric designs (Vellutino et al., 1975a) or letters in unfamiliar orthographies (i.e., Hebrew; Vellutino et al., 1975b). This pattern indicates that it is the linguistic features of letters and words that contribute to the reversal errors, rather than a general visual-spatial processing problem. The widespread misunderstanding regarding the causal role of letter reversals in dyslexia can be understood in the context of the common logical error of inferring causation from correlation. While children with dyslexia can reverse letters, and sometimes do so beyond developmentally typical windows, letter reversals are not a cause of their reading problems.

The myth of “backwards reading” is particularly pernicious because it leads to a fundamental misunderstanding of the cause of the disorder. The myth also interferes with best practices for assessment and treatment. For example, in our clinical experience, this myth has led to delayed referral for psychoeducational assessment because a child is not suspected of having dyslexia if they are not reversing letters. Misunderstanding of the causal factors can also delay access to evidence-based structured literacy interventions (Castles et al., 2018), while pursuing ineffective visual interventions (Pennington, 2008, 2011; American Academy of Pediatrics, 2009; Fletcher and Currie, 2011). Efforts to educate teachers, parents, and medical professionals about the true underlying causes of dyslexia continue through national professional associations (i.e., American Academy of Pediatrics, 2009, 2014) and non-profit foundations such as the International Dyslexia Association (i.e., Fletcher and Currie, 2011; Pennington, 2011), but it is clear that more work is needed to reach the public on this issue.

A recent study on the persistence of this dyslexia myth, among other misconceptions about the brain and learning, highlights the magnitude of this misunderstanding (Macdonald et al., 2017). Macdonald et al. (2017) examined endorsement of the dyslexia myth in a United States sample that recruited educators (*N* = 598), individuals with self-reported high neuroscience exposure (*N* = 234), and the general public (*N* = 3045). Results revealed surprisingly high rates of endorsement of the dyslexia myth: general public (76%), educators (59%), and individuals with high neuroscience exposure (50%). Strikingly, this sample had an over-representation of graduate level training and was recruited through a citizen science website on the brain and learning (testmybrain.org), so it is likely that the Macdonald et al. (2017) study actually *under*estimated prevalence of this myth. Overall, the results of this study were in line with similar findings from educator samples recruited from the United Kingdom (Dekker et al., 2012; Simmonds, 2014) and the Netherlands (Dekker et al., 2012), indicating a persistent and widespread misunderstanding about the causes of dyslexia.

Not only is this dyslexia myth prevalent among educators and even those with a neuroscience background, it is prevalent in higher education as well (Betts et al., 2019). Findings from an international study of awareness of neuromyths among higher education faculty, instructors, and instructional designers across education, psychology, and neuroscience disciplines (*N* = 929) revealed that the dyslexia myth was endorsed by nearly 80% of respondents. Specifically, Betts et al. (2019) found that the dyslexia myth (“a primary indicator of dyslexia is seeing letters backwards”) was endorsed at rates of 77% by higher education instructors, 76% by instructional designers, and 73% by administrators. Only a myth about the Mozart effect (“listening to classical music increases reasoning ability”) was endorsed at a higher rate than the dyslexia myth, at rates of 84% by higher education instructors, 89% by instructional designers, and 87% by administrators.

### Neuromyths

In the past decade, the dyslexia myth of backwards reading has been studied in relation to a larger family of misconceptions about the brain and learning, termed “neuromyths” (Della Sala, 2007; Lilienfeld et al., 2010). A growing number of recent studies have examined these myths in educators across the globe, from preschool to higher education teachers (see Betts et al., 2019), with a more limited number of research studies examining neuromyths in the general public (Macdonald et al., 2017). Macdonald et al. (2017) found a clustering of “classic” neuromyths (items related to learning styles, dyslexia, the Mozart effect, the impact of sugar on attention, right-brain/left-brain learners, and using 10% of the brain), such that the dyslexia myth was often endorsed by the same individuals who endorsed other neuromyths. This clustering of misconceptions about the brain and learning suggests that the dyslexia myth is associated with a larger group of neuromyths, raising the question of whether addressing these misconceptions by providing information about basic neurocognitive processes may ameliorate neuromyth beliefs as well as the dyslexia myth.

The past decade of research aimed at identifying misconceptions about the brain and learning, particularly among educators, also has focused on how these myths arise and why they persist (Pasquinelli, 2012; Howard-Jones, 2014). Howard-Jones (2014) argued that the most persistent neuromyths endorsed across PK12 through higher education are functions of “cultural distance” between neuroscience and education, tracing persistent myths about the brain and learning as germinating from “seeds of confusion,” “cultural conditions”, and biased distortions of scientific data (pp. 817–819). Similarly, Pasquinelli (2012) identified three processes about neuromyths’ origins as (1) distortions of scientific facts, (2) obsolete offspring of scientific hypotheses, or (3) outgrowths from misinterpretations of experimental results. In the case of the dyslexia myth, the origins of this myth can be found in obsolete ideas stemming from previously held scientific hypotheses. While this explains the origins of the myths, strategies for dispelling these myths must be responsive to the “cultural conditions” and “seeds of confusion” that have maintained these myths over time (Howard-Jones, 2014), requiring further education about updated models of dyslexia, with prominent contributions from neuroscience.

### Dispelling the Dyslexia Myth

Altogether, recent findings about endorsement of the dyslexia myth (Macdonald et al., 2017; Betts et al., 2019) suggest that it is one of the most common and persistent myths held by PK12 and higher education teachers. This is particularly concerning as teachers play a key role in identifying children who are at risk for dyslexia and helping to guide their families to appropriate assessment and intervention. Moreover, recent research indicates that higher education instructors, who are training the next generation of teachers, seem to be perpetuating this myth. What is needed to make progress on the dyslexia myth is further education about updated models of dyslexia, including current models from education, psychology, and neuroscience. Confusion about the causes and consequences of dyslexia coupled with lack of access to current scientific information about the brain and learning might be addressed through integrated and accessible scientific information available through online learning modules.

An initial step toward improved professional development in dispelling the dyslexia myth is to understand the extent to which existing publicly available information about dyslexia (i.e., online modules) address the dyslexia myth. Thus, the purpose of this narrative review is to identify and analyze publicly available online dyslexia learning modules to determine the extent to which they address the dyslexia myth. A secondary goal is to provide a content synthesis of existing online dyslexia learning modules in order to direct attention to existing resources for professional development.

## METHOD

### Definitions, Literature Search, and Inclusion Criteria

This section describes our procedure for identifying online dyslexia learning modules featured in this review. It includes definitions, search strategies, inclusion criteria, and coding procedures.

#### Online Learning Module

An online learning module is a “standalone educational unit or functional division of a course that provides materials with stated learning outcomes that may include, but not be limited to, topic-specific content (text, audio, video), discussions, assignments, and assessments (formative, summative) in a logical and sequential order offered through online, hybrid, or onsite formats” (Betts et al., in press). Another key feature of an online learning module is the structuring of targeted content for participants, such that they learn information sequentially before they may proceed further within the module.

#### Literature Search

We conducted our search of online learning modules using the Google search engine to identify current, online, open-access, dyslexia learning modules in English. Google search is identified as the most popular search engine globally, as well as an important source of governmental and institutional reports (Hagstrom et al., 2015). We defined open-access as being publicly available in terms of full or partial access to module content. The module search was completed in a 1-month period (May, 2018) and the findings were constrained by the following criteria.

#### Search Terms and Inclusion Criteria

The following search terms were used individually to identify the first 100 search results: dyslexia module, dyslexia teaching module, dyslexia learning module, and dyslexia educational module. These search terms were used individually and then with the search phrase “state department of education” (i.e. “dyslexia module” and “dyslexia module and state department of education”). The search phrase “state department of education” was added as a modifier to the search terms above to identify websites created by state governmental departments of education. We also specifically searched federal and state technical assistance centers (e.g., CEEDAR, IRIS), but these sources did not include online dyslexia modules that met our eligibility criteria. Our inclusion criteria were based on the characteristics of available online dyslexia learning modules and included: (a) availability, (b) learning outcomes, and (c) credibility.

#### Availability

Availability refers to whether an online learning module was publicly available on the internet in an open-access format. To be considered “available”, the module needed to be displayed within the first 100 search results from the searches described above, provide either full or partial access to content and be free of charge. The decision to limit the results to the first 10 pages (100 results) was based on the premise that to be available, a current, online, open-access module would display within the first 100 search results. Otherwise, it was unlikely to be found by potential users. Another selection criterion was open access. Within our initial pool of results, we could not access three modules contained within the first 100 search results without monetary exchange; thus, we included only those that were freely available to the public.

#### Learning Outcomes

Learning outcomes identify targeted knowledge that users acquire by completing the module. Learning outcomes are intended to focus the user on the application of knowledge and to guide feedback (e.g., assessment). In order to be considered for inclusion in this review, modules had to include stated learning outcomes.

#### Credibility

We defined a credible source as authorship/source content from an advocacy or government agency such as international, national, and regional dyslexia organizations, as well as federal and state departments of education, verified private companies and federal technical assistance organizations. We excluded any module from a source that could not be verified for credibility (i.e., website created by parent, student presentation, etc.).

#### Search Results

Our initial search for online dyslexia modules using Google yielded over 553,000 results. Using the inclusion criteria described above, including limiting results to the first 10 pages (100 results), we identified 14 online dyslexia modules. Each of the 14 identified modules met criteria of being freely available to the public (i.e., partial or full access to content), providing learning outcomes, and being published through a credible source. We verified the publication sources of each of the 14 modules for legitimacy and validity of claims and alignment with the current science of dyslexia.

#### Module Content Coding

We followed a thematic analysis process (Braun and Clarke, 2006, 2020) for identifying and coding content categories and thematic disciplinary domains. Reflexive thematic analysis is a recursive process involving movement back and forth between different sequential phases of the process. The phases of our analytic process were as follows. First, we familiarized ourselves with the module content. Second, we thoroughly outlined each module’s content and used inductive coding to generate content topics that were present across modules. This list of content topics served as a baseline for the development of the content codes used in this review. We categorized this baseline list of content codes according to disciplinary domains (Education, Neurocognition, and Policy). We then checked the content codes according to each domain for viability, definitions, and naming conventions. Two trained research assistants double-coded each module’s content codes and domains and compared results. Initial agreement for content categorization and domains was 85% with disagreements resolved through conferencing and review of the modules.

A total of 18 content topics in the domains of Education, Neurocognition, and Policy were coded for the 14 identified learning modules (see Table 1). Education-related topics on dyslexia included *age differences, assessment, characteristics* (e.g., decoding skills), *definition, interventions*, and *prevalence rates*. Neurocognition-related topics included *brain patterns, double-deficit hypothesis, dyslexia myth about backwards reading, genetic factors, neuroplasticity*, and *sex differences*. Policy topics included *environmental correlates* (e.g., socioeconomic status, literacy background) and *policy and practice implications*. Several topics were identified as belonging to multiple domains. For example, *cognitive factors* (e.g., processing speed, verbal working memory), *comorbidity, orthographic and auditory processing*, and *phonological processing*, as discussed in the modules, spanned Education and Neurocognition domains, while *environmental correlates* and *practice and policy implications* spanned Education and Policy domains. We coded *environmental correlates* within the Policy domain because module content pertaining to environmental factors such as socioeconomic status and home literacy background was presented in relation to state and federal policies.

## RESULTS

Findings are organized by descriptive and comparative characteristics of the 14 identified online dyslexia learning modules. First, we provide descriptive information on each of the modules. Next, we present comparative information and frequency of coded topics within and across the online dyslexia learning resources.

### Author, Source, and Disciplinary Domains

Table 2 describes each of the 14 identified online dyslexia modules by author, source, and percentage of topics identified within disciplinary domains. Module sources included state and federal educational agencies, advocacy organizations, and for-profit companies. Four of the 14 module sources were from advocacy organizations such as Learning Disabilities Association, LD Online/Reading Rockets, International Dyslexia Association (IDA), and British Dyslexia Association (BDA). Five of the 14 sources were federal and state educational agencies such as National Center for Learning Disabilities and Virginia Department of Education. Two of the modules were authored by educational agencies (South Carolina Department of Education, Open University) in partnership with advocacy organizations (e.g., IDA). Two module sources (Nessy Learning, Neuron Learning) were from for-profit-companies; and one module (IDA/Hoot) was a partnership between a non-profit organization (IDA) and a for-profit company (Hoot Education). None of the modules stated that specific background knowledge or experience was required, indicating that they were aimed more generally to users seeking an initial level of understanding of dyslexia, including professionals, caregivers, and individuals with dyslexia. See Appendix A for a complete listing of the 14 identified modules along with their website names and locations. Table 2 includes the mean percentage of Education, Neurocognition, and Policy-related topics covered across the 14 modules.

### Module Duration, Presentation Format, and Interactivity

Descriptive information on each of the identified online dyslexia modules by the characteristics of duration, presentation format, and interactivity is presented in Table 2. Duration refers to the recommended or designated time frame that was specified for users to complete the learning module, as confirmed by our research team. Module duration reflects the extent to which a user can self-pace their learning of content and corresponds with levels of interactivity. Although we did not identify module presentation, duration, or format as inclusion criteria, these characteristics appeared to influence the extent to which modules were accessible and interactive.

#### Duration

The completion time for all 14 modules ranged from 30 min to 8 h. The mean length of completion time across the modules was between 2 and 4 h, based on suggested times and our research team’s interaction experiences. Over half (*n* = 8) of the modules had completion times below 2 h. Two modules had completion times between 2 and 4 h. Four modules required more than 4 h to complete.

#### Presentation Format

Our findings reflect the significance of presentation format as influential to how content is processed and learned for different users. Thus, it is included in our descriptive findings. Presentation format refers to the use of graphic and technological platforms such as webinars, PowerPoints, 3d modeling, animation, and video formats to represent content, and to structure and guide participants through content learning.

The primary presentation format of modules was webinar. Module webinars relied on the use of visual and auditory supports such as animation, PowerPoint, voice-over, and video to promote users’ engagement. The primary presentation format of two modules was PowerPoint; and five modules relied on video presentation format with PowerPoint and animation embedded within. For example, Module 4 (LD Online/Reading Rockets) included a series of videos combining brain images, voice-over, and lecture to explain dyslexia’s biological origins.

#### Interactivity

Interactivity refers to the user’s engagement with the content through the module’s presentation format (e.g., clicking on textboxes, graphics, etc.). Interactivity includes the method of feedback that the module provided (e.g., quiz), the type of feedback (e.g., corrective), as well as the amount of feedback (e.g., explanations, further resources). Interactivity also includes the extent to which users are active learners, rather than passive recipients of presented information.

Two of the reviewed modules included short summative quizzes ranging from 5 to 10 questions (Modules 5 and 14). Two other modules provided users with key points on summary slides at the completion of different sections of the modules (Modules 6 and 13). Although all modules were self-paced to the extent that a user could control the start and stop of the module presentation, each was structured in a static, chronological order that required a user to complete all previous sections before advancing to the next part of the module. Therefore, none of the modules allowed for exploration of content (e.g., clicking or navigating through sections of the module independently).

### Dyslexia Module Coded Topics

Table 3 provides frequency data for each coded topic’s occurrence across the 14 modules, as well as the frequency of the 18 dyslexia topics within each module. The most frequently occurring topics across modules were *characteristics* (100%), *phonological processing* (100%), *cognitive factors* and *definition* (both 93%). Among the least frequently occurring topics were the *dyslexia myth about backwards reading* and *double-deficit hypothesis*, which occurred in only 14%, or 2 out of 14 modules.

Table 3 also provides information on percentage of topics identified within each of the 14 modules. Modules 8 (Open University) and 6 (Neuron Learning) covered the most topics, 16 out of 18 (89%); followed by Module 13 (NCLD/Understood), addressing 15 out of 18 topics (83%). Module 4 (LD Online, 2014) covered the fewest topics (6 out of 18, or 33%).

#### Education Topics

Education topics were pedagogical in nature and supported teachers, educational professionals, and parents to identify characteristics and to cultivate a supportive learning environment for individuals with dyslexia. The most frequently occurring Education-related topic was *characteristics*, which was included in all 14 modules, followed by *definition*, which was included in which 13 out of 14 modules (93%). The topic of *interventions* was covered in 86% of modules, followed by *age differences* and *assessment* (79%), and *prevalence rates* (64%).

#### Neurocognition Topics

The focal point of Neurocognition topics was the neurobiological basis of dyslexia. The topics of *brain patterns* and *genetic factors* were addressed in 6 out of 14 modules (43%). The topic of *neuroplasticity* was addressed in 4 out of 14 modules (36%), followed by *sex differences* (21%) in 3 out of 14 modules. The least frequently occurring topics were *double-deficit hypothesis* and *dyslexia myth about backwards reading*, which were identified in 2 out of 14 modules (14%).

#### Education-Neurocognition Topics

Topics that spanned both Education- and Neurocognition were among the most frequently occurring content categories. For example, *phonological processing* was addressed in 100% of modules, followed by *cognitive factors* (93%). *Comorbidity* and *orthographic and auditory processing* were addressed in 64% (9 out of 14) of the modules.

#### Education-Policy Topics

Only two policy topics were addressed across the 14 modules. Both topics were also relevant to education. Seventy-one percent (10 out of 14) of the modules included information on *practice and policy implications*, while only four out of 14 modules (29%) contained information on *environmental correlates*.

## DISCUSSION

We investigated publicly available online dyslexia learning modules to understand the extent to which these resources address the dyslexia myth regarding backwards reading. A surprising finding from our review was that only 2 of the 14 modules (Modules 10 and 13) contained information that directly addressed the most prevalent myth about dyslexia regarding “backwards reading.” While these two modules covered a range of education- and neuroscience-related topics pertaining to dyslexia, neither addressed the dyslexia myth within the context of a broader coordinated neuroscience primer focused on reading development to explain how or why the myth was false. While both modules included information on key dyslexia topics such as phonological processing, orthographic processing, auditory processing, and brain activation patterns, these topics were not presented in a coordinated way to dispel the dyslexia myth. In other words, the two online dyslexia learning modules that addressed the dyslexia myth did so by verbally stating it was a myth rather than by providing explanations from integrated neuroscience content on reading development and evidence-based interventions. Future modules may find it useful to provide explanatory information through broader discussion of the brain, cognition, and reading, which would provide a more complete understanding of dyslexia’s underlying etiology and the key role of evidence-based interventions for dyslexia.

### Module Format: Duration, Presentation, Accessibility, and Interactivity

The online learning modules varied by length and presentation type, with the majority focusing on the phonological and orthographic difficulties from speech-language and educational perspectives. One of our observations was in noting the wide variability across modules in terms of duration, presentation, and interactivity.

Duration was an important consideration in the context of accessibility and interactivity. Duration varied widely across the 14 modules, with most completion times exceeding 1 h. The length of time required to complete a learning module could influence the extent to which users completed or attained learning outcomes. Additionally, the lengthy duration of most of the modules did not provide for intermittent feedback (i.e., summative or performative), a key feature of interactivity.

Along with module duration, presentation format was a contributing factor to modules’ accessibility and interactivity. For instance, most modules used a webinar format as their technology platform, with varying visual and auditory presentation modes (PowerPoint, graphics, voice-over, and video) to convey information and to address learning outcomes. Graphics, animations, and 3d images, however, were used much less frequently to convey complex brain-behavior relationships, as compared to the use of PowerPoint slides with accompanying text descriptions and voice-overs, which required a relatively high level of receptive vocabulary (oral and written) as well as text reading skill. In addition to the accessibility of modular content, interactivity was influenced by presentation format, insofar as none of the modules allowed users to guide their own learning or to depart from the predetermined static order of the content presentation. As well, only a few modules provided users with corrective feedback for monitoring their understanding or comprehension of content during the presentations; only two modules provided formative feedback such as summary points at the conclusion of topics within the module.

Taken together, characteristics such as duration, presentation format, and interactivity of the available modules indicate that the content of many dyslexia learning modules may not be fully accessible to users for these reasons. Future studies could examine whether dyslexia learning modules that are shorter in duration, self-directed, and that utilize engaging visual representations of cognitive neuroscience concepts related to dyslexia lead to users’ improved knowledge of dyslexia. These enhancements to current modules could include summative and formative feedback on users’ mastery of topics pertaining to dyslexia, its etiology, and evidence-based instructional practices in an interactive and accessible format.

### Module Content: Education, Neurocognition, Education-Neurocognition, and Policy Topics

Our content analysis of online dyslexia modules revealed that most topics were focused across Education and Neurocognition disciplinary domains, followed by topics that spanned Education and Neurocognition. The most frequently occurring Education topics centered on pedagogical information such as assessment, characteristics, definition, as well as strategies and interventions at various ages and grade-levels. Dyslexia topics spanning Education and Neurocognition domains included cognitive factors, comorbidity with other learning disorders, orthographic and auditory processing, and phonological processing. Fewer modules addressed Neurocognition topics related to underlying etiology of dyslexia (e.g., double-deficit hypothesis), brain activation patterns, and neuroplasticity of the brain.

It is important to note that while all 14 online learning modules contained at least two neurocognition-related topics, only 2 (14%) modules (Module 10 and Module 13) presented information that directly addressed the dyslexia myth of backwards reading. Out of the three modules with the highest number of neurocognition-related topics (Modules 6, 8, and 13), only Module 13 included text stating that the dyslexia myth is false. This pattern suggests that the dyslexia myth is rarely addressed in online learning modules and this applies even to modules that have a neurocognitive focus. Moreover, the two modules that did address the myth did so without information from neuroscience to discredit the myth. They simply stated in text format that the myth is false rather than showing how or why it is false. To improve upon this, existing modules could address the dyslexia myth through presentation of integrated neuroscience content focused on the underlying etiology of dyslexia and the role of evidence-based interventions for dyslexia.

### Implications for Access to Dyslexia Content Learning Through Online Modules

Implications from the results of our review of online dyslexia learning modules point to the need for improved access to the basic neuroscience concepts underlying dyslexia, which could be useful for dispelling the dyslexia myth of backwards reading. For example, content focused on the reading brain and its disruptions in dyslexia could support improved understanding of the basis for dyslexia. Understanding that disruptions are in regions implicated in language and complex cognition, and not basic visual processes, could help correct the misunderstanding that dyslexia is based on reading backwards.

Additionally, our review highlights the need for improved access to content through self-directed and interactive presentation formats that are shorter in duration (less than 1 h), providing intermittent and varied feedback (e.g., formative, summative, corrective), and integrating graphic and visual formats with verbal (written and spoken) text to convey complex concepts about the nature of dyslexia and its interdisciplinary scientific basis to users. It is also important to note that many users who engage with this type of module may not have prior knowledge or experience in educational neuroscience, making the scaffolded learning environment even more critical to users’ understanding and retaining knowledge about dyslexia’s neurocognitive and linguistic underpinnings.

Another implication for the development of online dyslexia learning modules to promote increased accessibility to its scientific basis is in the type of content provided to users. It is remarkable that no modules address the dyslexia myth from an integrated educational neuroscience perspective. Current modules focus on definitions, characteristics, and interventions, but do not integrate educational neuroscience with evidence-based reading practices. In order to better understand the reading process, neurocognition topics such as the interactivity and development of brain regions associated with reading skill acquisition (e.g., reading network involving tempo-parietal junction and visual word form area, see Norton et al., 2015 for a discussion) could support an increasingly comprehensive understanding of reading development and disorders. As a result, the biggest drawback of the currently available dyslexia learning modules is the lack of interdisciplinary scientific content focused on reading development and evidence-based practices for dyslexia spanning education, psychology, and neuroscience fields.

While it is not necessary to have a neuroscience background to understand why the dyslexia myth about backwards reading is incorrect, it is the case that non-evidence-based treatments for dyslexia draw on brain myths to proliferate. Moreover, we have found in previous work that individuals who believe the dyslexia myth are also more likely to believe other neuromyths (Macdonald et al., 2017; Betts et al., 2019). Because the dyslexia myth and other misunderstandings about the brain seem to be clustering together and might even be mutually reinforcing, we suggest that it is important to understand the dyslexia myth in the context of these broader misunderstandings. Future research should examine whether interdisciplinary training modules can dispel the dyslexia myth for teacher-educators and practitioners. Additionally, it is important to examine whether changes in beliefs impact assessment and intervention practices for children with dyslexia. The effect of such a knowledge transmission approach remains an open question as we acknowledge that “debunking” messages are not always effective in countering misinformation (Chan et al., 2017).

Future studies also could explore whether differences exist in how the dyslexia myth is addressed in modules aimed at the general public as compared to those designed as part of a formal professional teaching credential, highlighting the need for practitioner responsibility in dispelling the dyslexia myth through evidence-based and scientifically grounded explanations of reading development and disorders.

In summary, a potentially comprehensive approach to dispelling the dyslexia myth could include a primer on basic neuroscience concepts underlying reading development, which would also address neuromyths more generally (e.g., those related to brain laterality, auditory and visual learning modalities).

### Limitations

This review is limited by some constraints. Only modules in English could be evaluated by the research team, so we cannot comment on resources that might be available in other languages. The use of Google search presents potential limitations based on our geographic location, which we did not account for in this study. Another limitation was that modules had to be available openly and indexed by Google, which could have limited the total number of identified modules in our analysis. A commonly reported limitation of using Google search in systematic reviews is that it could lead to personalization of results (Piasecki et al., 2018). We addressed this issue by logging out from all Google accounts. Although Google has identified limitations, it seemed apt for the purposes of our narrative content review focused on easily accessible public resources (e.g., Piasecki et al., 2017, 2018). Future research could examine the extent to which differences exist between openly available and restricted access online dyslexia learning modules.

It is also the case that the dyslexia myth is addressed in popular press articles, blogs, websites, and other online resources that are not part of learning modules. For example, the Association for Psychological Science (APS) maintains a page addressing the dyslexia myth, among others (Association for Psychological Science [APS], 2019) and prominent online venues have created neuromyth quizzes that include the dyslexia myth (Oakes, 2017). Robust websites such as International Dyslexia Association [IDA]’s (2020) *Decoding Dyslexia* are dedicated to increasing educational interventions for dyslexia and aiming to raise dyslexia awareness among educators, policy makers, and caregivers. Likewise, a great deal of information on the history, causes, characteristics, diagnostics, and neuroscience research on dyslexia can be found on Wikipedia (Anis et al., 2019). While these more static presentations are helpful for reaching the general public, we believe that educators working with students with dyslexia can benefit from a more interactive learning module that dispels the dyslexia myth and delivers high quality content about the underlying cognitive-linguistic and neurobiological basis of dyslexia.

### Directions for Research and Practice: Dyslexia Learning Module Featuring Neuroscience

Recognizing the limitations of existing modules, we see a need for a comprehensive online dyslexia learning module that includes educational neuroscience content focused on reading development in an accessible format to users outside of the neuroscience community (e.g., pre-service and in-service educators, families, school administrators, higher education faculty), with the goal of bringing the interdisciplinary science of dyslexia to a wider audience. We argue that access to high quality and integrated science content through an interactive learning format such as an online learning module should be part of a comprehensive strategy for dispelling the persistent dyslexia myth about backwards reading. Secondarily, there exist important questions of whether educational misconceptions are related to one another and whether remediation of one can influence endorsement of another. Such an online learning module with an educational neuroscience orientation to dyslexia might help to dispel related neuromyths (i.e., we only use 10% of our brain, right brain-left brain misconceptions, etc.). Our hypothesis is that when educators better understand the cognitive-linguistic (e.g., weaknesses in phonological processing, orthographic and auditory processing) and neurobiological correlates of dyslexia, they will gain a deeper understanding of dyslexia and be better equipped to screen for dyslexia and to select and deliver evidence-based treatments. Certainly, we aim to curtail ineffective visual treatments which are based on a misunderstanding of the disorder as “backwards reading.”

Higher education could utilize such a module to improve educational neuroscience knowledge among faculty and instructors (see Betts et al., 2019) as well as among pre-service teachers. Potentially, schools of education could provide introductory neuroscience instruction at the undergraduate and graduate levels so that educators possess a basis for evaluating pedagogical approaches purportedly based in educational neuroscience. There is clearly a proliferation of such “brain-based learning” claims in education and it is important that educators have foundational skills to evaluate them. One way to provide this introductory content could be by discussing common misconceptions about the brain and learning (i.e., the dyslexia myth and other neuromyths) and their roots as well as practices that are outgrowths of these misconceptions. This discussion could serve as a foundation for conveying the complexity of the brain and learning. Such discussion might fit well into existing educational psychology courses.

In summary, our search for online dyslexia learning modules identified 14 open-access and publicly available modules from verified credible sources. Only two explicitly addressed the dyslexia myth, which is a pervasive misunderstanding that continues to interfere with efficient screening and effective treatment. The apparent gap in the professional development resources suggests the need for the development of a learning module that provides access to high quality integrated content focused on cognitive-linguistic and neurobiological basis of dyslexia. This learning module could address the myth of backwards reading through interactive, scaffolded, and lexile-controlled content (i.e., readability) with scientific explanations of brain-behavior relationships involved in reading, and could potentially promote increased accessibility and target misconceptions about the brain and reading.

## Figures and Tables

**TABLE 1 |:** Coded dyslexia module topics, definitions, and disciplinary categories.

Topic	Definition	Discipline
1. Age differences	Differences in manifestations of dyslexia across age groups, including symptoms of dyslexia; how age-group differences serve as a foundation for educators to teach students at different ages.	• Education
2. Assessment	Standardized tools that compare students with dyslexia to a pre-established baseline, for identifying differences in phonological awareness, sound/symbol correspondence, alphabetic knowledge, decoding/encoding skills, and rapid automatized naming (RAN) skills.	• Education
3. Brain patterns	Structural and functional brain imaging technology that reveals neural differences between readers with and without dyslexia.	• Neurocognition
4. Characteristics	Dyslexia is associated with learning challenges in sound-symbol correspondence leading to problems with decoding. Secondary consequences include reading comprehension and writing difficulties.	• Education
5. Cognitive factors	Verbal working memory and processing speed are commonly associated with dyslexia.	• Education, neurocognition
6. Comorbidity	Dyslexia is comorbid with learning disorders such as dysgraphia, dyscalculia, ADHD, and developmental language disorder.	• Education, neurocognition
7. Definition	Dyslexia is a specific learning disorder that is neurobiological in origin and is connected to the phonological component of language.	• Education
8. Double-deficit hypothesis	A deficit in both RAN and phonological awareness is associated with the most severe cases of dyslexia.	• Neurocognition
9. Dyslexia myth	Misconception that dyslexia is caused by seeing letters backwards.	• Neurocognition
10. Environmental correlates	Environmental factors influence learning and dyslexia, as well as the impact of social issues (resources, opportunities, etc.) on dyslexia.	• Education, policy
11. Genetic factors	Genetics (genes, heritability) influence dyslexia; extent to which dyslexia runs in families.	• Neurocognition
12. Interventions	Structuring educational interventions focused on language and literacy learning techniques, and the use of technology to assist learning.	• Education
13. Neuroplasticity	The brain’s ability to reorganize itself through the creation of new neural connections; in this case, the ability of brain activity to change after intervention.	• Neurocognition
14.Orthographic and auditory processing	The roles of orthographic and auditory processing in the brain (e.g., how we see and hear words/sounds, how both are involved in dyslexia).	• Education, neurocognition
15. Phonological processing	Phonological processing is involved in development of phonological awareness skills, developed through phonemic awareness and grapheme-phoneme correspondence.	• Education, neurocognition
16. Practice and policy implications	Practice and policy regulations and implications related to federal and state government laws supporting educational interventions and procedures for identification of students with dyslexia.	• Education, policy
17. Prevalence rates	Prevalence of dyslexia across age, language, ethnicity/race, and sex.	• Education
18. Sex differences	Differences in the manifestation of characteristics of dyslexia among males and females.	• Neurocognition

**TABLE 2 |:** Author, source, percentage of topics in disciplinary domains, and descriptive characteristics of online dyslexia modules.

Module	Author	Source			Percentage in disciplinary domains				Descriptive characteristics		
		Education agency	Advocacy group	For-profit company	Education (Ed)	Neurocognition	Ed-neurocognition	Ed-policy	Duration (minutes)	Presentation	Format
1	Addressing Dyslexia (Scotland)		✓		0.50	0	0.30	0.20	120	Webinar	Self-paced
2	British Dyslexia Association		✓		0.42	0.17	0.33	0.08	300	Webinar	Self-paced
3	International Dyslexia Association/Hoot Education		✓	✓	0.50	0.30	0.10	0.10	60	Powerpoint	Self-paced
4	LD Online/reading rockets		✓		0.17	0.17	0.50	0.17	30	Videos	Self-paced
5	Nessy learning			✓	0.57	0	0.43	0	180	Webinar Videos	Self-paced quiz
6	Neuron learning			✓	0.31	0.31	0.25	0.13	70	Powerpoint	Self-paced
7	New Hampshire Department of Education	✓			0.43	0.29	0.21	0.07	180	Webinar	Self-paced
8	OpenLearn Open University	✓	✓		0.38	0.25	0.25	0.13	480	Webinar	Self-paced
9	Pemberton Township Schools (Burlington, NJ)	✓			0.56	0	0.33	0.11	120	Videos	Self-paced
10	South Carolina Department of Education	✓	✓		0.50	0.08	0.33	0.08	60	Videos	Self-paced
11	State Government of Victoria, Australia	✓			0.63	0	0.38	0	120	Webinar	Self-paced
12	Texas Dyslexia Identification Academy	✓			0.63	0	0.38	0	360	Webinar	Self-paced
13	National Center for Learning Disabilities/Understood	✓			0.40	0.27	0.27	0.07	60	Webinar	Self-paced
14	Virginia Department of Education	✓			0.60	0	0.30	0.10	40	Webinar	Self-paced quiz
Mean					0.47	0.14	0.31	0.09	156		

**TABLE 3 |:** Frequency of topics’ occurrence within and across dyslexia modules.

Topic	Occurrence in modules														% of Modules
	1	2	3	4	5	6	7	8	9	10	11	12	13	14	
1. Age differences	✓	✓	✓		✓	✓	✓	✓		✓	✓		✓	✓	79%
2. Assessment	✓	✓	✓				✓	✓	✓	✓	✓	✓	✓	✓	79%
3. Brain Patterns			✓	✓		✓	✓	✓					✓		43%
4. Characteristics	✓	✓	✓	✓	✓	✓	✓	✓	✓	✓	✓	✓	✓	✓	100%
5. Cognitive factors	✓	✓		✓	✓	✓	✓	✓	✓	✓	✓	✓	✓	✓	93%
6. Comorbidity		✓				✓	✓	✓	✓	✓	✓	✓	✓		64%
7. Definition	✓	✓	✓		✓	✓	✓	✓	✓	✓	✓	✓	✓	✓	93%
8. Double-Deficit Hypothesis						✓		✓							14%
9. Dyslexia Myth										✓			✓		14%
10. Environmental correlates	✓		✓			✓		✓							29%
11. Genetic factors		✓	✓			✓		✓		✓			✓		43%
12. Interventions	✓		✓		✓	✓	✓	✓	✓	✓	✓	✓	✓	✓	86%
13. Neuroplasticity		✓	✓			✓	✓						✓		36%
14. Orthographic/auditory processing	✓	✓		✓	✓	✓		✓		✓			✓	✓	64%
15. Phonological processing	✓	✓	✓	✓	✓	✓	✓	✓	✓	✓	✓	✓	✓	✓	100%
16. Practice and policy implications	✓	✓		✓		✓	✓	✓	✓	✓			✓	✓	71%
17. Prevalence rates		✓				✓	✓	✓	✓	✓		✓	✓	✓	64%
18. Sex differences						✓	✓	✓							21%

Total Percent	56%	69%	56%	33%	39%	89%	72%	89%	50%	72%	44%	44%	83%	56%	

## APPENDIX A

**TABLE A1 |:** Dyslexia online learning module authors and websites.

Module	Author and website
1	Addressing Dyslexia (Scotland) http://addressingdyslexia.org/what-dyslexia
2	British Dyslexia Association https://www.bdadyslexia.org.uk/dyslexia
3	International Dyslexia Association http://media.wix.com/ugd/ace015_03a2e27cd8384e5d9a6db46849395e9f.pdf
4	LD Online https://www.youtube.com/watch?v=G2vWNGv9W3E&index=1&list=PLLxDwKxHx1yL88wZUEmFk7GK1BShr35UY
5	Nessy Learning https://www.nessy.com/us/product/professional-development/
6	Neuron Learning http://www.neuronlearning.com/2017-dyslexia-research-and-remediation/
7	New Hampshire Department of Education https://www.education.nh.gov/instruction/dyslexia/index.htm
8	OpenLearn Open University https://www.open.edu/openlearn/education-development/education/understanding-dyslexia/content-section-0?active-tab=description-tab
9	Pemberton Township Schools (Burlington, NJ) https://www.youtube.com/watch?v=o--DO0aAVHw
10	South Carolina Department of Education https://ed.sc.gov/districts-schools/special-education-services/additional-information-and-assistance/dyslexia-and-other-reading-disorders/dyslexia-module-1-what-is-it-and-what-do-we-know-about-it/
11	State Government of Victoria, Australia http://www.education.vic.gov.au/school/teachers/teachingresources/discipline/english/reading/Pages/default.aspx
12	Texas Dyslexia Identification Academy https://www.texasgateway.org/course/texas-dyslexia-identification-academy-dyslexia-foundations
13	National Center for Learning Disabilities https://www.understood.org/en/learning-attention-issues/child-learning-disabilities/dyslexia/understanding-dyslexia#item1
14	Virginia Department of Education http://www.doe.virginia.gov/teaching/licensure/dyslexia-module/story.html
